# A Note on the Iterative MRI Reconstruction from Nonuniform *k*-Space Data

**DOI:** 10.1155/2007/24727

**Published:** 2007-03-13

**Authors:** Tobias Knopp, Stefan Kunis, Daniel Potts

**Affiliations:** ^1^Institute of Mathematics, University of Lübeck, 23538 Lübeck, Germany; ^2^Department of Mathematics, Chemnitz University of Technology, 09107 Chemnitz, Germany

## Abstract

In magnetic resonance imaging (MRI), methods that use a non-Cartesian grid in *k*-space are becoming increasingly important. In this paper, we use a recently proposed implicit discretisation scheme which generalises the standard approach based on gridding. While the latter succeeds for sufficiently uniform sampling sets and accurate estimated density compensation weights, the implicit method further improves the reconstruction quality when the sampling scheme or the weights are less regular. Both approaches can be solved efficiently with the nonequispaced FFT. Due to several new techniques for the storage of an involved sparse matrix, our examples include also the reconstruction of a large 3D data set. We present four case studies and report on efficient implementation of the related algorithms.

## 1. INTRODUCTION

The raw data for magnetic resonance imaging (MRI) is measured in *k*-space, the domain of spatial frequencies, where non-Cartesian sampling schemes like spiral or radial scans have received much attention. In contrast to the use of the computationally efficient fast Fourier transform (FFT) for the reconstruction from Cartesian grids, the more general sampling trajectories ask for the so-called nonequispaced FFTs. On the other hand, iterative image reconstruction algorithms play an important role in modern tomographic systems [1]. Recently, iterative image reconstruction in combination with the nonequispaced FFT has been applied to data on spiral *k*-space trajectories [2] and in the presence of field inhomogeneities [3]. Efficient reconstruction procedures for sensitivity encoding with arbitrary *k*-space trajectories were proposed in [4]. Its authors present methods that combine the gridding principles with the conjugate gradient scheme, but mention the long computation times due to their nonoptimised preliminary software.

In this paper, we use a similar method, compare the gridding approach [5] and an approach based on an implicit discretisation [6], where we focus for the reader's convenience on a simplified signal equation. It turns out that the iterative solution of the latter approach resembles the gridding method in its first iteration. Both reconstruction problems are easily solved by our mature software package [7] for the nonequispaced fast Fourier transform (NFFT) [8, 9]. For readers not familiar with the NFFT, we suggest to read the appendix of this paper first. The publicly available implementation easily allows for the efficient treatment of large 3D data sets by the use of new sparse matrix storage techniques [10]. Moreover, we compare different density compensation weights arising from the discretisation of the underlying integrals. We present numerical results, based on the Shepp-Logan phantom as well as on data acquired by an MR scanner.

The outline of this paper is as follows: Section 2 gives a brief introduction to the theory of Fourier transform image reconstruction and unifies the two considered approaches in MRI. We suggest the conjugate gradient method for the reconstruction problem and show that the solution is efficiently computed by the iterative use of the nonequispaced FFT. Subsequently, Section 3 presents the used *k*-space trajectories, considers sampling density compensation, and introduces the tested simulated and acquired data sets. Section 4 shows our numerical tests emphasising the reconstruction quality with respect to the number of iterations and different density compensation weights. Moreover, we give detailed information on the computational requirements of the suggested scheme. Finally, a short discussion of our results is contained in Section 5 and an introduction to the nonequispaced FFT might be found in the appendix.

## 2. THEORY

Given a trajectory **k** = **k**(*t*), the relation between the MR signal *s* during the readout and the object *p* can be modeled by the simplified signal equation

(1) $$s(\text{k})=\int_{{\mathbb{R}}^{3}}p(r){\text{e}}^{2\pi \text{i}rk}\text{d}r.$$

In the following, we describe two different approaches. For convenience, let the available samples in *k*-space be contained in the shifted unit cube, that is, **k** ∈ [−1/2,1/2)^3^, and the field of view be restricted to Ω_N_ ⊂ [−*N*_1_/2,*N*_1_/2) × [−*N*_2_/2,*N*_2_/2) × [−*N*_3_/2,*N*_3_/2), where **N** = (*N*_1_, *N*_2_, *N*_3_)^Τ^ ∈ 2ℕ^3^. Then, the discretisation of integral (1) on equispaced points leads to

(2) $$s(k)\approx \tilde{s}(k):=\sum_{r\in I_{\text{N}}^{3}}p(r){\text{e}}^{2\pi \text{i}rk},$$

where *I* ^3^_N_ := {−*N*_1_/2,…, *N*_1_/2 − 1} × {−*N*_2_/2,…, *N*_2_/2 − 1} × {−*N*_3_/2,…, *N*_3_/2 − 1}. Thus, the unknown object *p* is given *implicitly* by (2). The authors of [6] call this the “inverse model.”

A second approach uses the Fourier inversion theorem $p(r)=\int_{{\mathbb{R}}^{3}}s(k){\text{e}}^{-2\pi \text{i}rk}\text{d}k$ first. The discretisation of this integral leads to

(3) $$p(r)\approx \overset{˜}{p}(r):=\overset{M-1}{\underset{j=0}{\sum }}s(k_{j}){\text{e}}^{-2\pi \text{i}rk_{j}}w_{j},$$

where *w*_*j*_ are weights, which compensate for local variations of the sampling density. Here, the unknown object $p\approx \tilde{p}$ can be computed *explicitly* .

The important difference between (2) and (3) is that the former is discretised in the image domain with pixels on a uniform grid and hence with unit weighting coefficients and the latter is an integral discretised in *k*-space with nonuniform samples and specific weights. We reformulate problem (2) and (3) in matrix vector notation and denote the vector of the given measurements by **s** := (*s*(**k**_*j*_) )_*j*=0, …,*M*−1_ ∈ ℂ^*M*^, the reshaped vector of the unknown object by **p** :=(*p*(**r**))_**r**∈*I*^3^_N__ ∈ ℂ^*N*_1_× *N*_2_× *N*_3_^, the density compensation matrix by **W** :=diag(*w*_*j*_)_*j*=0, …, *M*−1_, and the nonequispaced Fourier matrix by

(4) $$A:={\left({\text{e}}^{2\pi \text{i}rk_{j}}\right)}_{j=0,\ldots ,M-1;\;r\in I_{\text{N}}^{3}},$$

whereas **A** ^𝖧^ denotes its adjoint (conjugate transpose).

The gridding approximation (3) is easily computed by one matrix vector multiplication

(5) $$\overset{˜}{p}=A^{\text{𝖧}}Ws.$$

The adjoint NFFT takes *𝒪*(|*I* ^3^_N_| log |*I* ^3^_N_| + *M*) floating point operations for this task.

Slightly more involved, the reconstruction problem (2) is solved by the method of least squares and hence consists in solving the weighted normal equation of first kind

(6) $$A^{\text{𝖧}}WAp=A^{\text{𝖧}}Ws$$

for the unknown vector **p**. In contrast to [6], we include density compensation weights also for the implicit discretisation since this is more natural with respect to the “continuous residual” in *k*-space and has been proven to be better conditioned in [11]. From the mathematical point of view, (6) is solved most efficiently by the conjugate gradients (CG) method (cf. [12, page 288]). We prefer to solve (6) by a factorised variant of conjugated gradients, where the two multiplications with the (adjoint) nonequispaced Fourier matrix per iteration are computed by the NFFT. This scheme is denoted by CGNR, N for “Normal equation,” and R for “Residual minimisation” (cf. [13]). Note that the CG method applied directly to (6) as suggested in [4] generates the same sequence of approximations in exact arithmetic, but the CGNR approach is considered to be more stable with respect to round-off errors (cf. [14, Section 7.1]). In summary, we suggest Algorithm 1.

Remarkably, this algorithm resembles an optimised gridding solution after one iteration. More formally, let the weighted residual norm ||**r**||^2^_W_ := **r** ^𝖧^ **Wr** be given. Then, the first iteration obeys

(7) $$p_{1}=\arg\underset{p=\alpha \tilde{p}}{\min}∥s-Ap{∥}_{\text{w}},$$

since for the first residual **r**_1_ = **s** − **Ap**_1_ holds the perpendicular condition

(8) $$r_{1}^{𝖧}WA\overset{˜}{p}={\left(A^{𝖧}Ws\right)}^{𝖧}\overset{˜}{p}-\frac{{\overset{˜}{p}}^{𝖧}\overset{˜}{p}}{{\left(A\overset{˜}{p}\right)}^{𝖧}WA\overset{˜}{p}}{\left(A\overset{˜}{p}\right)}^{𝖧}WA\overset{˜}{p}=0.$$

In other words, the gridding solution $\overset{˜}{\text{p}}$ in (5) is scaled such that its residual is minimised and we do not consider a gridding approach separately anymore.

Moreover, note that the proposed scheme should be stopped as soon as the current residual ||**s** − **AP**_*l*_||_**w**_ drops below the discretisation error or the level of noise in the measurements **s**. For our first evaluation, we terminate the suggested algorithm after a fixed number of iterations.

## 3. METHODS

We are concerned with the reconstruction from data acquired by an MR scanner as well as the reconstruction quality and the usage of time and memory resources for simulated data. Algorithm 1 has been tested with MR measurements of a physical phantom by the Philips Achieva 1.5T device. Here, the sampling scheme consists of 36 equidistant radial trajectories with *M* = 7 557 120 points in total, whereas the reconstructed image contains 256 × 256 × 36 = 2 359 296 voxels.

**Figure 1:**
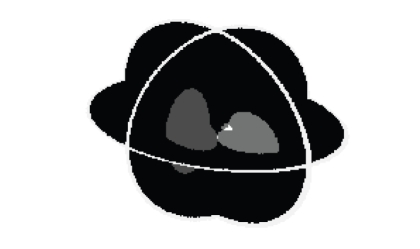
Slice plot of our 3D-Shepp-Logan phantom.

Moreover, we compare different reconstructions for simulated MR data. We use two 3D-Shepp-Logan phantoms of sizes 256 × 256 × 36 and 128 × 128 × 128 (2 097 152 voxels) as shown in Figure 1. Comparison is done with respect to the number of iterations, sampling schemes, and density compensation weights. The simulated MR data is computed by a 3D-NFFT using the following *k*-space trajectories. Note that all but the first trajectory are of a special 2D ⊗ 1D type, that is, they consist of a stack of 36 equidistant planes where the *M*_1_ points within each are distributed accordingly. In these cases, the first two coordinates of a *k*-space point **k** = (*k*_1_, *k*_2_, *k*_3_)^⊤^ form the 2D-point $\overset{˜}{k}={\left(k_{1},k_{2}\right)}^{\text{⊤}}\in {\left[-1/2,1/2\right)}^{2}$.

(i) (3D-RADIAL) The only 3D-*k*-space trajectory, which is not of 2D ⊗ 1D type, is given by
  (9) $$k_{p,q,r}=\frac{r+1}{\sqrt{2}R}{\left(\cos\phi_{p}\sin\theta_{q},\sin\phi_{p}\sin\theta_{q},\cos\theta_{q}\right)}^{\text{⊤}},$$
  where *ϕ*_*p*_ = 2*π* *p*/*P*, *p* = 0,…, *P* − 1, θ_*q*_ = *π*(2*q* + 1)/2*Q*, *q* = 0,…, *Q*− 1, and *r* = 0,…, *R* − 1. Furthermore, we restrict this set to the unit cube ( −1/2, 1/2)^3^. Choosing *P* = *Q* = *R* = 160 in our experiments yields a total number of *M* = 3 398 033 points in *k*-space.
(ii) (RADIAL) Popular also within computer tomography is the 2D-radial trajectory
  (10) $${\tilde{k}}_{p,r}={\left(-1\right)}^{r}\left(\frac{r}{R}-0.5\right){\left(\cos\;\frac{\pi p}{P},\sin\;\frac{\pi p}{P}\right)}^{⊤}$$
  with *p* = 0,…, *P* − 1, *r* = 0,…, *R* − 1. We set *P* = 410 and *R* = 512 yielding a total number of *M* = 36*M*_1_ = 36*PR* = 7 557 120 points in *k*-space.
(iii) (SPIRAL) This 2D-*k*-space trajectory is given by one Archimedean spiral, that is,
  (11) $${\overset{˜}{k}}_{j}=\frac{\sqrt{j}}{2\sqrt{M_{1}}}{\left(\cos\omega_{j},\sin\omega_{j}\right)}^{\text{⊤}},$$
  where $\omega_{j}=(8\pi /5)\sqrt{j}$, and *j* = 0,…,*M*_1_ − 1. We have chosen *M*_1_ = 65 536 yielding a total number of *M* = 36 *M*_1_ = 2 359 296 points in *k*-space.

**Figure 2:**
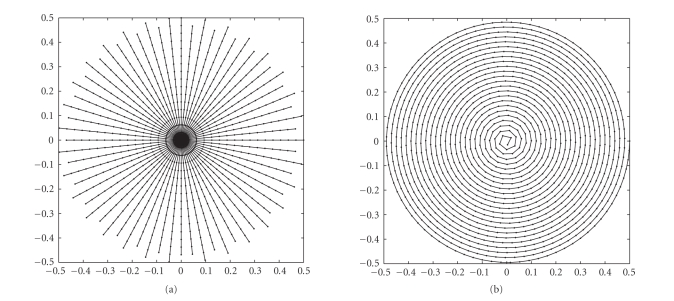
Typical *k*-space trajectories, (a) RADIAL (*P* = *R* = 32) and (b) SPIRAL (*M*_1_ = 1024).

Examples of the trajectories are shown in Figure 2. Note that each proposed sampling scheme obeys a Nyquist rate near the origin of the *k*-space. Nevertheless, all sampling sets violate the Nyquist criteria in their periphery since there exist boxes larger than the reciprocal field of view 1/*N*_1_ × 1/*N*_2_ × 1/*N*_3_ containing no single sampling point **K**_*j*_.

In the following, different weights **W** in (6) are used to take into account the local sampling density in *k*-space. We propose formulations

- with no weights, that is, **W** being the identity,
- with analytic weights in the case of the 3D-RADIAL trajectory, given by
  (12) $$w_{p,q,r}=\frac{r+1}{R}\sin\frac{\pi (2q+1)}{2Q},$$
- with approximate weights computed in a very cheap way by counting the number of sample points in the 256 × 256 box tesselation of the *k*-space [ −1/2, 1/2]^2^, and
- with weights obtained as the area of the Voronoi cell
  (13) $${\text{Ω}}_{j}=\{\overset{˜}{k}\in {\left[-\frac{1}{2},\frac{1}{2}\right)}^{2}:∥\overset{˜}{k}-{\overset{˜}{k}}_{j}{∥}_{2}\leq \underset{l=0,\ldots ,M-1}{\min}∥\overset{˜}{k}-{\overset{˜}{k}}_{l}{∥}_{2}\}$$
  around each sample point ${\tilde{k}}_{j}$; see also [15, 16].

It was pointed out, for example, in [16], that the use of Voronoi weights as a measure of the local sampling density is a very reliable and general technique. For the gridding approach (3), a detailed discussion of sampling density compensation for sampling sets violating the Nyquist criteria is given in [17].

## 4. NUMERICAL RESULTS

Algorithm 1 was implemented as part of the NFFT library [7]. All tests were implemented in Matlab&C and tested on an AMD Athlon XP 2700 +, 2GB memory, SuSe-Linux, kernel 2.4.20−4GB-athlon, FFTW3.0.1, and NFFT2 (Kaiser-Bessel window function with cut-off parameter *m* = 6 and oversampling factor *σ* ≥ 1.5). Besides a couple of representative examples, our web page on this project http://www.tu-chemnitz.de/∼potts/projects/mri collects a more thorough set of tests as pointed out in the subsequent examples. In particular, this includes animated graphics showing the progress during the iterations or slicing through the 3D data set.

**Numerical Example 1**

**Figure 3:**
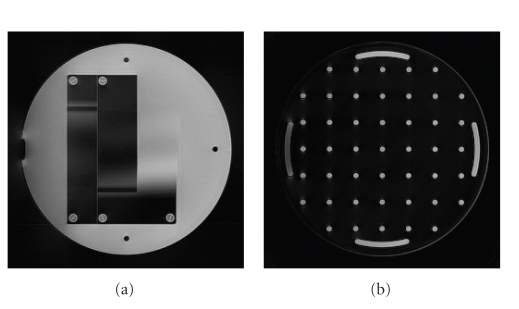
Two slices of the reconstruction from MR measurements with Voronoi weights after one iteration.

First of all, we apply Algorithm 1 to MR measurements taken by the Philips Achieva 1.5T device. Figure 3 shows the result after one iteration, where we used Voronoi weights for sampling density compensation.

**Numerical Example 2**

We compare the reconstruction quality with respect to the three different *k*-space trajectories and sampling density compensation weights. For the 2D ⊗ 1D type of the trajectories, we reconstruct each slice separately and compute the overall reconstruction by 256 ^2^ regular 1D-FFTs of length *N*_3_ = 36 within the third component. Table 1 shows the normalised root-mean-square error

(14) $$\text{RMS}(\overset{˜}{p},p):=\frac{∥p-\overset{˜}{p}{∥}_{2}}{∥p{∥}_{2}},$$

where $\overset{˜}{\text{p}}$ is our reconstruction and **p** denotes the original image of size 128 × 128 × 128 for the 3D-RADIAL trajectory and of size 256 × 256 × 36 for all others.

Additionally, we computed 30 iterations for the 3D-RADIAL example which yields a RMS of 0.3309 and 0.2104 for no weights and analytic weights, respectively. A first hint on the stability of the reconstruction process with respect to noisy data is given in the last line of Table 1. We show the RMS for the reconstruction from simulated SPIRAL data which is perturbed by 20 percent standard normally distributed noise.

**Numerical Example 3**

**Figure 4:**
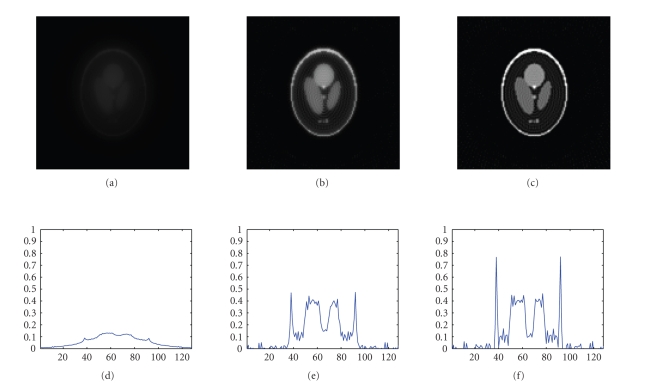
Sixty-fourth slice of the phantom (top) and the profile of the Sixty fourth row of this slice (bottom) with 3D-RADIAL data and * analytic weights* (from left to right after one, five, and ten iterations).

We present some reconstructions our algorithm achieves. The main purpose of the 3D-RADIAL example is to demonstrate that the NFFT-based reconstruction is straightforward. Figure 4 shows the 64th slice and the profile of the 64th row of this slice after one, five, and ten iterations for analytic weights. The same experiment using no weights is presented on our web page.

**Figure 5:**
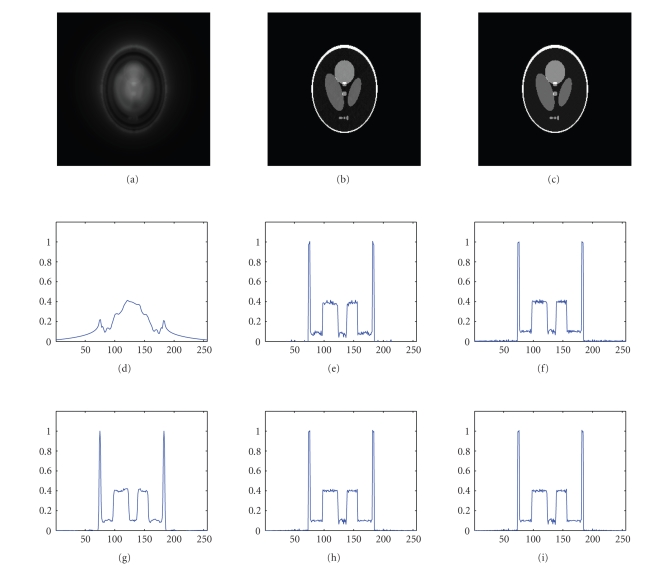
18th slice of the phantom (top) and the profile of the 128th row of this slice (middle) after *one iteration* with RADIAL data: from left to right, without weights, with approximative weights and with Voronoi weights. The same profile is shown after *ten iterations* (bottom).

Furthermore, we show the 18th slice and the profile of the 128th row of this slice after one and ten iterations for different weights and the 2D ⊗ 1D *k*-space trajectory RADIAL in Figure 5. The same experiment using the SPIRAL trajectory and an interleaved spiral trajectory is presented on our web-page.

**Numerical Example 4**

In the last test, we measured CPU time and used memory of our algorithms with respect to the size of the reconstruction problem *N*, that is, the size of the reconstructed image is *N* × *N* × 36 and the total number of SPIRAL *k*-space samples is 36*N* ^2^. We compare Algorithm1exploiting the 2D ⊗ 1D type of the trajectory with Algorithm 1 using the full 3D NFFT. Both algorithms produce almost the same sequence of reconstructions.

The optimisation of the computing time is done by different NFFT flags which affect the last step of the NFFT. In this last step, we compute by *𝒪*(*M*) floating point operations the matrix vector product with the sparse matrix **B** as defined in the appendix, (A.2). We propose different methods for the precomputation and storage of this matrix that basically trade main memory for computation speed. We were able to store all nonzero entries of the matrix **B** together with their row and column index by the flag PRE-FULL-PSI in all 2D ⊗ 1D tests; see [18] for a similar approach. A lossless compressed form, flag PRE-PSI, is used in the 3D-tests up to *N* = 256. Finally, we use a lossy compressed version which is independent of the actual trajectory, uses a lookup table, and linear interpolation for the entries of the sparse matrix (PRE-LIN-PSI) in the 3D-tests for *N* = 512, see the manual to [7, 10] for details. Table 2 shows our results for the Shepp-Logan phantom, the SPIRAL trajectory, and Voronoi weights. The measured CPU-times grow as expected like *N* ^2^ log*N*. Clearly, the method based on the 2D ⊗ 1D model is faster, however our algorithms can handle arbitrary scattered data in *d* dimensions by storing a heavily compressed form of the sparse matrix **B**.

## 5. DISCUSSION

Magnetic resonance signals are measured in *k*-space, where magnetic field gradients determine the specific trajectories that form the sampling points. We have shown that the reconstruction from non-Cartesian grids is easily computed by means of the nonequispaced FFT where at least an approximate compensation of the non-uniform sampling density proves necessary for highly nonuniform sampling sets.

The gridding approach, based on an explicit discretisation, and the “inverse model” [6], based on an implicit discretisation, are unified and solved by Algorithm 1. In particular, the gridding solution is optimised in one iteration to minimise the residual, see (7), and further refined in subsequent iterations. We see that already the gridding method may lead to very good results in Table 1 and in Figure 5. However, using no density compensation weights gives reasonable results only if the trajectory covers the *k*-space uniformly as for the SPIRAL trajectory, see Table 1 “weights: none” and our web-page. Gridding without density compensation gives non satisfactory results for 3D-RADIAL and RADIAL trajectories as shown in the leftmost images of Figures 4 and 5. Including approximate weights improves the reconstruction quality, the best gridding results are obtained using the more expensive Voronoi weights. However, substantial improvement of the reconstruction quality is achieved during a small number of iterations for all used trajectories and weights. Furthermore, only the iterative method in conjunction with a reasonable approximation of weights gives an acceptable reconstruction from the 3D-RADIAL trajectory (cf. Figure 4 and our web page). Finally, we have compared the computation time and memory usage of the proposed algorithm for the 2D ⊗ 1D model and a full 3D model.

In summary, the implicit discretisation and its iterative solution generalises the gridding approach in a natural way and refines the image quality. Particularly, a poor gridding solution from a highly nonuniform trajectory with approximated density compensation weights is improved substantially by a few iterations of our scheme. We have shown that very efficient methods are available, which can be generalised to reconstruction methods for sensitivity encoding as proposed in [4] and to reconstruction methods in presence of field inhomogeneities [3, 19].

Note, however, that the computation times reported in Table 2 depend on the stopping criterion. Directions for our future research include the development of reliable stopping criteria for the iterative reconstruction and proven convergence rates when the Nyquist criteria is violated.

## Figures and Tables

**Algorithm 1:**
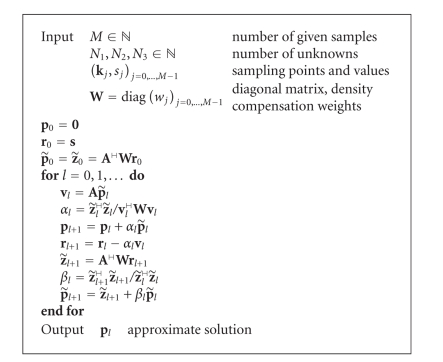
Conjugate gradients for normal equations (CGNR).

**Table 1:** RMS for different weights and *k*-space trajectories after 1, 2, 5, and 10 iterations.

Weights\iterations	1	2	5	10
3D RADIAL				

None	0.8281	0.7491	0.6482	0.5509
Analytic	0.6981	0.5685	0.3047	0.2146

RADIAL				

None	0.6458	0.5276	0.3025	0.1170
Approximation	0.1597	0.0773	0.0767	0.0764
Voronoi	0.0776	0.0775	0.0772	0.0769

SPIRAL				

None	0.1658	0.0908	0.0769	0.0767
Approximation	0.1686	0.0864	0.0773	0.0768
Voronoi	0.1360	0.0812	0.0781	0.0779

SPIRAL with noise				

Voronoi	0.1444	0.0937	0.0971	0.1010

**Table 2:** CPU time and memory usage for different iteration numbers (no.) and sizes *N* of the *k*-space data and the reconstructed phantom.

	CPU time in seconds				MByte
*N*\no.	1	2	5	10	

2D ⊗ 1D trajectory					

512	118.95	178.04	355.08	650.55	691
256	29.24	43.55	87.21	159.71	172
128	6.56	9.84	19.73	35.78	43
64	1.45	2.16	4.31	8.00	10

3D trajectory					

512	11 422	17 114	34 189	62 650	1952
256	1865.9	2800.4	5603.3	10 274	1244
128	452.31	679.28	1344.9	2454.4	311
164	89.24	133.72	267.18	489.59	77

## APPENDIX

In recent years, the nonequispaced fast Fourier transform (NFFT) has attracted much attention due to the fact that it generalises the FFT for arbitrary sampling geometries. Common names for these algorithms are *nonequispaced FFT* [8, 9, 20], *nonuniform FFT* [21], *generalised FFT* [22], or *unequally spaced FFT* [23]. Its accuracy is adjusted to the practical requirements by an oversampling factor and a cut-off parameter, whereas no dependency on the sampling points occurs. In contrast, the inverse NFFT can be computed with a CG-type algorithm utilising one NFFT and one adjoint NFFT per iteration and its reconstruction error depends strongly on the sampling geometry and used compensation weights. In what follows, we briefly describe the NFFT, the adjoint NFFT, and the inverse NFFT. These methods are implemented in our public software package [7].

Nonequispaced FFT The first problem to be addressed here can be regarded as a matrix vector multiplication. For a finite number of given Fourier coefficients fr^ ∈ ℂ, **r** ∈ *I* ^*d*^_N_ := { −*N*_1_/2,…, *N*_1_/2 − 1} × {−*N*_2_/2,…, *N*_2_/2 − 1} × ⋯ × { − *N*_*d*_/2,… *N*_*d*_/2 − 1} with **N** := (*N*_1_,…, *N*_*d*_)^⊤^ ∈ 2ℕ^*d*^, one wants to evaluate the trigonometric polynomial

(A.1) $$f(x):=\sum_{r\in I_{\text{N}}^{d}}{\hat{f}}_{r}{\text{e}}^{2\pi \text{i}rx}$$

at given points **x**_*j*_ ∈ [ −1/2, 1/2)^*d*^, *j* = 0,…, *M* − 1. In matrix vector notation, see also (4), this reads as f=Af^  . Fast approximate matrix vector multiplication with **A** and its adjoint matrix **A** ^𝖧^ are known as NFFT and adjoint NFFT, respectively. A unified approach for this task was suggested in [8, 9] and is based on the factorisation **A** ≈**BFD** and the use of a simultaneously in space and frequency localised window function *φ*. The diagonal matrix **D** collects the inverse of the sampled Fourier transform of the window function D=[0,diag(1/φ^(r))r∈INd,0]⊤. Subsequently, we apply an oversampled Fourier matrix **F** = **F**_*σ***N**_, where *σ* > 1 denotes a chosen oversampling factor. Finally, we multiply with the sparse matrix **B** = (*b*_*j*,**1**_)_*j*=0,…,*M*−**1**,**I**∈*I*^d^_N__ having for some cut-off parameter *m* ∈ ℕ at most (2*m* + 1)^*d*^ nonzero entries per row, that is,

(A.2) $$b_{j,1}=\varphi (x_{j}-\frac{1}{\sigma N}),-\frac{m}{\sigma N}\leq x_{j}-\frac{1}{\sigma N}\leq \frac{m}{\sigma N}.$$

The relation between speed and accuracy of the algorithm was initially investigated in [8, 9, 22, 23]. The accuracy of the NFFT increases exponentially with the cut-off parameter *m* for each fixed oversampling factor *σ* > 1. The total number of floating point operations is of order *𝒪* (|*σ* *I* ^*d*^_*N*_| log |*σ* *I* ^*d*^_*N*_| + *m* ^*d*^ *M*) . In various papers, different window functions *φ* for the NFFT were considered, for example, a Gaussian pulse tapered with a Hanning window in [24], Gaussian kernels combined with sinc kernels in [25], and special optimised windows in [24, 26]. Furthermore, special approaches based on scaling vectors [27], based on minimising a discrete norm of certain error matrices [28], or based on min-max interpolation [21] are proposed. We remark that the NFFT and its adjoint can be realised with a broad class of these window functions yielding the same accuracy by changing the cut-off parameter *m*. In particular, the obtained reconstruction quality in MRI is fairly independent of the particularly chosen window function. However, numerical results in [20, 21, 28] show that the Kaiser-Bessel window function provides the best accuracy for a fixed cut-off parameter *m*, that is, number of nonzeros per row of the matrix **B**. Moreover, the library adapts over a wide class of available memory through the so-called flags; see, for example, Numerical Example 4. The manual to [7, 10] present details for a suitable choice of the window function, the cut-off parameter, and the oversampling factor with respect to accuracy, speed, and memory usage. The adjoint algorithm, that is, the evaluation of the sums${\hat{g}}_{r}:=\sum_{j=0}^{M-1}f_{j}{\text{e}}^{-2\pi \text{i}rx_{j}}$, **r** ∈ *I* ^*d*^_N_, can be computed by the matrix vector multiplication with **A** ^𝖧^, where we obtain a fast algorithm by using the same factorisation **A** ^𝖧^ ≈ **D** ^⊤^ **F** ^𝖧^ **B** ^⊤^ . It was already pointed out in [8, 29], that the gridding method is simply a fast algorithm for the multiplication of the matrix **A** ^𝖧^ with a vector. Including a sampling density compensation yields the following gridding algorithm, see also [26, 25]:

- sampling density compensation, that is, multiplication with a diagonal matrix **W**;
- approximation to an oversampled Cartesian grid, that is, multiplication with the matrix **B** ^⊤^;
- inverse fast Fourier transform, that is, multiplication with **F** ^𝖧^;
- roll-off correction, that is, multiplication with **D** ^⊤^.

Note that the role of the window function, appearing in the matrices **D** and **B**, and the sampling density compensation, appearing in **W**, should not be mixed up. We stress again that the NFFT and the adjoint NFFT (gridding) are algorithms that realise the matrix vector multiplications in a fast and efficient way.

Inverse nonequispaced FFT The aim of the inverse NFFT is to construct a trigonometric polynomial *f*, respectively, its Fourier coefficients fr^, **r** ∈ *I* ^*d*^_N_, such that *f*(**x**_*j*_) approximates given values *y*_*j*_ ∈ ℂ , that is,

(A.3) $$y_{j}\approx f(x_{j})=\sum_{r\in I_{\text{N}}^{d}}{\hat{f}}_{r}{\text{e}}^{2\pi \text{i}rx_{j}},j=0,\ldots ,M-1.$$

In matrix vector notation, this reads asAf^≈y. We propose to solve this system of linear equations by the method of least squares with sampling density compensation weights *w*_*j*_ > 0, that is,

(A.4) $$\underset{\hat{f}}{\min}∥y-A\hat{f}{∥}_{\text{w}}^{2}=\underset{\hat{f}}{\min}\sum_{j=0}^{M-1}w_{j}|y_{j}-f(x_{j}{)|}^{2},$$

where **W** := diag(*w*_*j*_)_*j*=0,…,*M*−1_. The iterative solution of this problem is addressed in [11, 30] by solving the explicitly formed Toeplitz system **A** ^𝖧^ **WA** f^ = **A** ^𝖧^ **Wy**. This method is known as ACT (adaptive weights, conjugate gradients, Toeplitz) and has been proven to be superior to previous established methods like POCS (projection onto convex sets) or the Landweber iteration (cf. [30]). The inverse NFFT applies the conjugate gradient method as well, but uses the NFFT instead of methods for Toeplitz matrices.
